# Supplementary material for: Modeling glioblastoma heterogeneity as a dynamic network of cell states
Source: Mol Syst Biol. 2021 Sep 16;17(9):e10105. doi: 10.15252/msb.202010105 (PMC8444284; doi:10.15252/msb.202010105)
Supplement: Supplementary file 6 — Source Data for Figure 5 [file MSB-17-e10105-s004.zip › Figure5A_sourcedata/GSEA_3017/nosigdb_stateD.GseaPreranked.1622795010397/LARSSON_STATE_4.html]

Details for gene set LARSSON\_STATE\_4[GSEA]

|  || Dataset | state23017 |
| Phenotype | NoPhenotypeAvailable |
| Upregulated in class | na\_neg |
| GeneSet | LARSSON\_STATE\_4 |
| Enrichment Score (ES) | -0.67750716 |
| Normalized Enrichment Score (NES) | -3.4620497 |
| Nominal p-value | 0.0 |
| FDR q-value | 0.0 |
| FWER p-Value | 0.0 |
Table: GSEA Results Summary

  

Fig 1: Enrichment plot: LARSSON\_STATE\_4      
 Profile of the Running ES Score & Positions of GeneSet Members on the Rank Ordered List

  

| PROBE | GENE SYMBOL | GENE\_TITLE | RANK IN GENE LIST | RANK METRIC SCORE | RUNNING ES | CORE ENRICHMENT || 1 | PTTG1 |  |  | 14 | 0.490 | -0.0001 | No |
| 2 | LSM5 |  |  | 123 | 0.274 | -0.0799 | No |
| 3 | BIRC5 |  |  | 130 | 0.271 | -0.0786 | No |
| 4 | MKI67 |  |  | 206 | -0.254 | -0.1326 | No |
| 5 | TUBB |  |  | 410 | -0.274 | -0.2882 | No |
| 6 | UHRF1 |  |  | 438 | -0.278 | -0.3034 | No |
| 7 | MCM10 |  |  | 515 | -0.287 | -0.3575 | No |
| 8 | E2F1 |  |  | 521 | -0.287 | -0.3550 | No |
| 9 | SIVA1 |  |  | 567 | -0.293 | -0.3842 | No |
| 10 | RFC2 |  |  | 607 | -0.302 | -0.4085 | No |
| 11 | MCM3 |  |  | 625 | -0.305 | -0.4152 | No |
| 12 | CHAF1A |  |  | 679 | -0.315 | -0.4503 | No |
| 13 | TMPO |  |  | 688 | -0.317 | -0.4495 | No |
| 14 | KIF4A |  |  | 706 | -0.320 | -0.4558 | No |
| 15 | MCM4 |  |  | 783 | -0.339 | -0.5087 | No |
| 16 | FANCI |  |  | 801 | -0.345 | -0.5145 | No |
| 17 | H2AFX |  |  | 810 | -0.347 | -0.5130 | No |
| 18 | CENPM |  |  | 818 | -0.349 | -0.5107 | No |
| 19 | MAD2L1 |  |  | 819 | -0.350 | -0.5028 | No |
| 20 | TK1 |  |  | 902 | -0.375 | -0.5597 | No |
| 21 | CDC45 |  |  | 1000 | -0.413 | -0.6276 | No |
| 22 | TPX2 |  |  | 1034 | -0.437 | -0.6440 | No |
| 23 | SMC2 |  |  | 1062 | -0.452 | -0.6554 | No |
| 24 | EZH2 |  |  | 1070 | -0.455 | -0.6507 | No |
| 25 | MND1 |  |  | 1083 | -0.461 | -0.6498 | No |
| 26 | ZWINT |  |  | 1099 | -0.467 | -0.6512 | No |
| 27 | ASF1B |  |  | 1133 | -0.498 | -0.6663 | Yes |
| 28 | PCNA |  |  | 1142 | -0.504 | -0.6613 | Yes |
| 29 | NUSAP1 |  |  | 1154 | -0.515 | -0.6584 | Yes |
| 30 | GMNN |  |  | 1155 | -0.516 | -0.6467 | Yes |
| 31 | DBF4 |  |  | 1157 | -0.520 | -0.6358 | Yes |
| 32 | RRM1 |  |  | 1166 | -0.534 | -0.6301 | Yes |
| 33 | BRCA1 |  |  | 1171 | -0.541 | -0.6211 | Yes |
| 34 | SGO2 |  |  | 1176 | -0.546 | -0.6119 | Yes |
| 35 | ORC6 |  |  | 1179 | -0.551 | -0.6011 | Yes |
| 36 | CENPU |  |  | 1182 | -0.555 | -0.5902 | Yes |
| 37 | DHFR |  |  | 1197 | -0.576 | -0.5883 | Yes |
| 38 | CKS1B |  |  | 1198 | -0.577 | -0.5753 | Yes |
| 39 | GTSE1 |  |  | 1210 | -0.602 | -0.5705 | Yes |
| 40 | CLSPN |  |  | 1212 | -0.605 | -0.5576 | Yes |
| 41 | KIF15 |  |  | 1213 | -0.610 | -0.5438 | Yes |
| 42 | CENPK |  |  | 1220 | -0.619 | -0.5346 | Yes |
| 43 | DUT |  |  | 1223 | -0.633 | -0.5219 | Yes |
| 44 | SMC4 |  |  | 1224 | -0.638 | -0.5075 | Yes |
| 45 | MYBL2 |  |  | 1225 | -0.639 | -0.4931 | Yes |
| 46 | CKS2 |  |  | 1226 | -0.641 | -0.4786 | Yes |
| 47 | SGO1 |  |  | 1246 | -0.691 | -0.4781 | Yes |
| 48 | UBE2T |  |  | 1256 | -0.719 | -0.4691 | Yes |
| 49 | HMGB2 |  |  | 1263 | -0.736 | -0.4572 | Yes |
| 50 | RAD51AP1 |  |  | 1264 | -0.741 | -0.4405 | Yes |
| 51 | TYMS |  |  | 1272 | -0.770 | -0.4287 | Yes |
| 52 | ATAD2 |  |  | 1275 | -0.775 | -0.4128 | Yes |
| 53 | PCLAF |  |  | 1281 | -0.828 | -0.3981 | Yes |
| 54 | ESCO2 |  |  | 1283 | -0.846 | -0.3797 | Yes |
| 55 | RRM2 |  |  | 1295 | -0.933 | -0.3674 | Yes |
| 56 | KPNA2 |  |  | 1297 | -0.972 | -0.3463 | Yes |
| 57 | FBXO5 |  |  | 1301 | -0.992 | -0.3263 | Yes |
| 58 | NCAPG |  |  | 1303 | -1.015 | -0.3041 | Yes |
| 59 | HIST1H1E |  |  | 1309 | -1.319 | -0.2783 | Yes |
| 60 | HIST1H1C |  |  | 1310 | -1.391 | -0.2469 | Yes |
| 61 | CDK1 |  |  | 1311 | -1.433 | -0.2146 | Yes |
| 62 | UBE2C |  |  | 1312 | -1.466 | -0.1815 | Yes |
| 63 | TOP2A |  |  | 1313 | -1.528 | -0.1470 | Yes |
| 64 | HIST1H1B |  |  | 1318 | -1.922 | -0.1067 | Yes |
| 65 | HIST1H1D |  |  | 1319 | -1.958 | -0.0625 | Yes |
| 66 | HIST1H4C |  |  | 1320 | -2.769 | -0.0000 | Yes |
Table: GSEA details [plain text format]

  

Fig 2: LARSSON\_STATE\_4: Random ES distribution      
 Gene set null distribution of ES for **LARSSON\_STATE\_4**
